# Global trends and spatial drivers of diabetes mellitus mortality, 1990-2019: a systematic geographical analysis

**DOI:** 10.3389/fendo.2024.1370489

**Published:** 2024-04-12

**Authors:** Zejia Xu, Jianheng Feng, Siyi Xing, Yin Liu, Yuting Chen, Jie Li, Yunhui Feng

**Affiliations:** ^1^ School of Geographical Sciences and Remote Sensing, Guangzhou University, Guangzhou, China; ^2^ Faculty of Innovation and Design, City University of Macau, Macao, Macao SAR, China; ^3^ Key Laboratory of Philosophy and Social Sciences in Guangdong Province of Maritime Silk Road of Guangzhou University (GD22TWCXGC15), Guangzhou, China; ^4^ Center for Interdisciplinary Health Management Studies, School of Physical Education & Sports Science, Guangzhou University, Guangzhou, China

**Keywords:** diabetes, risk factor, sustainable development goals, spatiotemporal variation, spatial regression

## Abstract

**Objective:**

Diabetes mellitus is the leading cause of death worldwide, and multiple risk factors associated with diabetes mortality.

**Methods:**

Employing spatial statistics, we characterized the spatial distribution and patterns of diabetes mortality, and revealed the spatial relationship between diabetes mortality and 11 socioeconomic and environmental risk factors at the country level, from 1990 to 2019.

**Results:**

Globally, significantly high rates of diabetes mortality were primarily clustered in countries with limited land areas or located on islands, such as Fiji, Kiribati, Eswatini, and Trinidad and Tobago. Countries with weaker economic independence are more likely to have higher diabetes mortality rates. In addition, the impact of socioeconomic and environmental factors was significant at the country level, involving health expenditure, number of physicians, household and ambient air pollution, smoking, and alcohol consumption. Notably, the spatial relationship between diabetes mortality and ambient air pollution, as well as alcohol consumption, showed negative correlations. Countries with high diabetes mortality rates generally had lower levels of ambient air pollution and alcohol consumption.

**Conclusion:**

The study highlights the spatial clustering of diabetes mortality and its substantial variation. While many risk factors can influence diabetes mortality, it’s also essential to consider the level of these factors at the country level. Tailoring appropriate interventions based on specific national circumstances holds the potential to more effectively mitigate the burden of diabetes mortality.

## 1 Introduction

Diabetes mellitus is one of the major disease burdens in global public health. People with diabetes not only have a range of metabolic disorder problems, but also have an increased risk of cancer and other non-communicable diseases (NCD), compared with the general population (1). Diabetes is one of the top 10 causes of death worldwide, which caused approximately 1.5 million deaths in 2019 (2, 3). Diabetes deaths account for 35.6% of deaths from NCD and 2.7% of deaths from all causes worldwide (2). The burden of diabetes has developed into an essential constraint on public health and economic development.

Diabetes, as a prominent global disease burden, has garnered substantial and focused attention in recent years (4). Previous studies have analyzed diabetes from diverse perspectives, including epidemiology (3), risk factors (3, 5), and social or economic burdens (6, 7). These investigations have provided a relatively comprehensive picture of the epidemiological process of diabetes and the impact of various external factors on diabetes (4, 8). However, spatial aspects are also worth paying attention to in diabetes spatial epidemiology research. These spatial pattern disparities in risk factors contribute to the spatial heterogeneity of risk factors, ultimately resulting in spatial heterogeneity in diabetes burden and diabetes-related health inequalities. Specifically, the heterogeneity in socioeconomic status is associated with inequalities in diabetes healthcare, leading to elevated diabetes deaths in countries with various incomes (6). Given that, investigating spatial patterns and heterogeneity is conducive not only to mitigating the disease burden of diabetes but also to narrowing the associated health inequalities.

To comprehend the spatial pattern and heterogeneity of diabetes more comprehensively, several issues continue to merit consideration and resolution. Notably, the majority of studies on diabetes traditionally treated areas as independent geographic units, often overlooking the interplay between areas (3, 9). In contrast, spatial statistics integrate spatial relationships into calculations, facilitating the direct exploration of the spatial distribution and dynamics of diabetes (9, 10). This approach also offers a more accurate assessment of how risk factors impact distinct geographic areas. Furthermore, in recent decades, the number of deaths due to diabetes has significantly increased in countries of varying regions or income levels, with significant variations in the underlying factors (11, 12). Globalization and economic development change are intricately correlated with the widespread prevalence of diabetes (4, 13). With globalization and economic development, multiple risk factors that directly or indirectly influence diabetes have undergone significant changes (13, 14). These factors encompass multiple macro aspects, such as healthcare services, lifestyle changes, environmental pollution, social well-being, and urbanization (14). Further, it is imperative to comprehensively illustrate the spatial heterogeneity of these factors at a broader geographic scale. Consequently, there is a vital necessity to investigate the long-term, global-scale spatial distribution and patterns of diabetes mortality, as well as the spatial relationship between social and environmental factors and diabetes mortality.

This study adopts a geographical epidemiology approach to reveal the spatial distribution, changes, patterns, and relationship of diabetes mortality. Utilizing spatial statistics and diabetes mortality data at the country level, it is possible to explore the spatial distribution and patterns of diabetes on a global scale, revealing regional clusters and disparities in diabetes mortality. The spatial statistics applied in this section encompass the Moran’s *I* statistics, and Getis-Ord *G_i_* ^*^. Furthermore, drawing from the Sustainable Development Goals (SDGs), this study collected 11 risk factors potentially associated with diabetes mortality. Through the application of two spatial relationship techniques, namely the Geodetector and spatial lag model, this study quantifies the spatial correlation and regression between spatial heterogeneity of diabetes mortality and these risk factors.

## 2 Methods

### 2.1 Data sources

Deaths due to diabetes were defined as death where diabetes is the underlying cause (15–18). These deaths included those caused by type 1 and type 2 diabetes, categorically identified as 5A10 and 5A11 according to the International Classification of Diseases (ICD) version 11 (19). The diabetes data for this study encompasses estimated age-standardized mortality rates of diabetes mellitus for 203 countries and territories from 1990 to 2019. The diabetes data were collected from the Global Health Data Exchange query tool (http://ghdx.healthdata.org/gbd-results-tool), maintained by the Institute for Health Metrics and Evaluation (IHME).

Accounting for differences in age structure, we utilized the age-standardized mortality rate to represent diabetes mortality in this study. The age-standardized mortality rate is calculated by applying the age-specific mortality rates observed within a particular population to the standard population’s age structure (20). This standard population typically represents a hypothetical population with a standard age distribution, here the World Standard Population. Adjusting for age in this manner enhances the accuracy of mortality rate comparisons between countries with different age structures.

There are two classifications of regions to measure diabetes mortality from geographic or economic perspectives. We have divided countries into 21 GBD regions based on geographic locations to highlight regional variations. Meanwhile, we have categorized countries into four groups based on World Bank income levels to highlight economic disparities across countries, including low-income countries (LICs), lower-middle-income countries (LMICs), upper-middle-income countries (UMICs) and high-income countries (HICs).

Diabetes mortality attributable to risk factors is based on the SDG indicators. SDGs provide a comprehensive framework of indicators closely related to sustainable development, such as health, environment, poverty, inequality, and well-being (2). In this study, we selected nine SDG indicators and two indicators related to the SDGs to contrast the framework of factors influencing diabetes mortality (**Table 1**). These factors were divided into three categories, namely health and healthcare, environment, development, and well-being.

**Table 1:** Summary of 11 risk factors for diabetes mortality.

Category	Risk factor	Interpretation	Unit	SDGs
Health and Healthcare	Per capita health expenditure	Current health expenditure per capita	1000$	SDG 1.a.2
	Universal health coverage	UHC Service Coverage Index reflects essential healthcare service coverage	%	SDG 3.8.1
	Number of physicians	Number of physicians per 1,000 people	Per 1,000	SDG 3.c.1
	High body-mass index	Proportion of the population exposed to a high body mass index	–	SDG 2.2.2
	Overweight in children	Overweight prevalence among children under 5 years of age	%	SDG 2.2.2
	Alcohol consumption	Per capita alcohol consumption for the population aged 15 and above	L	SDG 3.5.2
	Tobacco smoking prevalence	Estimate of current tobacco smoking prevalence	%	SDG 3.a.1
Environment	Household air pollution	The proportion of the population with primary reliance on polluting fuels and technologies for cooking	%	SDG 7.1.2
	Ambient air pollution	Concentrations of fine particulate matter (PM2.5)	–	SDG 11.6.2
Development and wellbeing	Urban population	The proportion of the population living in urban areas	%	–
	Human Development Index	Composite index reflecting a country’s health, education, and living standards	–	–

Given limitations in data availability, we utilized risk factors from 2019 for spatial relationship analysis. The data for the 11 risk factors in 2019 were collected from multiple sources. From the Global Health Observatory, the health dataset of the World Health Organization (WHO), we obtained the data for multiple risk factors, including per capita health expenditure, universal health coverage, number of physicians, overweight in children, alcohol consumption, tobacco smoking prevalence, household air pollution, ambient air pollution (21). Data for high body-mass index was obtained from IHME (22). Data for urban population was obtained from the United Nations World Urbanization Prospects (23). Data for the human development index was obtained from the United Nations Human Development Reports 2020 (24). To conduct spatial analysis, these data are at the country level, encompassing 203 countries and territories as possible.

### 2.2 Statistical analysis

#### 2.2.1 Spatiotemporal analysis

In spatiotemporal analysis, spatial statistical methods were employed to explore the spatial variation, temporal change, and spatial distribution pattern of diabetes mortality rates from 1990 to 2019, at the country level. We investigated the temporal trends for age-standardized mortality rates of diabetes from 1990 to 2019 by calculating the average annual percentage change (AAPC) along with 95% confidence intervals (CI) through a Joinpoint regression analysis. The AAPCs calculations were performed using the Joinpoint software (v5.0.2, https://surveillance.cancer.gov/joinpoint/), maintained by the U.S. National Cancer Institute. The Permutation Test (Monte Carlo Permutation) was selected to determine the significant level of AAPCs. Then, we employed Global Moran’s I to examine the global spatial autocorrelation of diabetes mortality rates from 1990 to 2019 (Moran, 1950), and Anselin local Moran’s *I* and Getis-Ord *G_i_* ^*^ was utilized to reveal and visualize the local spatial autocorrelation and distribution of diabetes mortality rates in 1990 and 2019. Spatial analyses, including Moran’s I statistics and Getis-Ord *G_i_* ^*^, were conducted in ArcGIS Pro (v3.0, ESRI, Redlands, CA, USA). The Compact Miller projection was employed for geographical visualization and spatial analyses on a global scale (25).

Global Moran’s *I* is a frequently used spatial statistical method for global spatial autocorrelation of geographical features. It characterizes the spatial patterns of geographical features by measuring feature locations and attribute values (26). We applied Moran’s I to determine the spatial patterns of diabetes mortality, exploring whether it is spatial clustering or dispersion. The equation for the calculation is as follows:


$$I=\frac{n}{\sum_{i=1}^{n}\sum_{j=1}^{n}w_{ij}}\frac{\sum_{i=1}^{n}\left(x_{i}-\bar{x}\right)\sum_{j=1}^{n}W_{ij}\left(x_{j}-\bar{x}\right)}{\sum_{i=1}^{n}{\left(x_{i}-\bar{x}\right)}^{2}}$$

where n is the total number of countries; $x_{i}$ and $x_{j}$ are diabetes mortality rates of countries *i* and *j* (where *i* ≠ *j*); $\bar{x}$ is the average over all locations of countries; $w_{ij}$ is the spatial weight between countries *i* and *j*. Spatial weight quantifies the spatial relationships or connectivity between different countries, and we used a distance-based spatial Weight in this study. The value of Moran’s *I* ranged from -1 to 1. A positive Moran’s *I* indicate spatial clustering of health indicators, while a negative Moran’s *I* suggest spatial dispersion. A Moran’s *I* equal to 0 implies a random distribution of health indicators. The *Z*-score and *P*-value provide statistical significance on the calculated Moran’s *I* using a 95% confidence level.

Anselin local Moran’s *I* is a spatial statistic used to detect local spatial autocorrelation in geographical features. It is designed to identify local clustering or dispersion patterns in space. Local Moran’s *I* reveal the spatial correlation between each geographic unit and its neighboring units by computing the spatial relationships and uncovering the local spatial structure around each location. The equation used for the calculation is as follows:


$$I_{i}=\frac{n}{W_{i}}\overset{n}{\underset{j=1}{\sum }}W_{ij}\left(x_{i}-\bar{x}\right)\left(x_{j}-\bar{x}\right)$$

where $I_{i}$ is the Moran’s *I* for country *i*. Local Moran’s *I* results can be categorized into four quadrants: high-high, high-low, low-high, and low-low. Specifically, high-high indicates a country and its neighboring countries both have high values, suggesting the local clusters of high values. High-low indicates a country has a high value, but its neighboring countries have low values, suggesting the local dispersal of high values. The *Z*-score and *P*-value also provide statistical significance for Local Moran’s *I*, corresponding to a 95% confidence level.

Following Anselin local Moran’s *I*, Getis-Ord *G_i_* ^*^ was employed to further reveal the significance of local clusters of geographical features. Getis-Ord *G_i_* ^*^ is a spatial statistic that analyzes local spatial patterns of geographical features. It determines whether the clustering pattern of features is high- or low-value concentration (27). The equation for the calculation is as follows:


$$G_{i}^{*}=\;\frac{\sum_{j=1}^{n}w_{ij}x_{j}-\bar{x}\sum_{j=1}^{n}w_{ij}}{\sum_{j}^{n}w_{ij}x_{j}}$$

where $w_{ij}$ is the spatial weight between countries *i* and *j*. Positive *G_i_* ^*^ indicates that country *i* is surrounded by high values mortality, and the country is regarded as a hot spot; negative *G_i_* ^*^ indicates that country *i* is surrounded by low values mortality, and the country is regarded as a cold spot. In addition, based on statistical significance, *G_i_* ^*^ can be categorized into four levels: high significance, middle significance, low significance, and not significance.

#### 2.2.2 Spatial relationship analysis

In relationship analysis, spatial analysis techniques were employed to quantify the spatial correlation and regression between diabetes mortality rates and risk factors in 2019. Initially, Geodetector was utilized to evaluate the individual contributions of risk factors to the spatial heterogeneity observed in diabetes mortality rates. Then, we fit the ordinary least squares (OLS) model to adjust the spatial lag model. Based on the OLS model, variance inflation factors (VIF) were computed for the risk factors to assess potential multicollinearity. Further, Lagrange multiplier (LM) and robust LM tests were conducted to determine the reliability of the spatial lag model and to mitigate against multiple hypotheses. Last, the spatial lag model was employed to investigate the spatial impact of risk factors on spatial heterogeneity of diabetes mortality rates. To objectively compare the performance of the OLS and spatial lag model, fit statistics such as *R* ^2^ and Akaike Information Criterion (AIC) statistics were employed (28).

The Geodetector is a spatial statistic for detecting spatial stratified heterogeneity (SSH) of spatial features (29). Unlike methods requiring linear hypotheses, the Geodetector offers a capacity to evaluate the individual impact of driving factors on spatial features. If the spatial distribution of diabetes mortality rates is similar to that of a given factor, then the distribution of diabetes mortality rates can be attributed to the factor. The equation for the calculation is as follows:


$$q=1-\frac{\sum_{h=1}^{L}N_{h}\sigma_{h}^{2}}{N\sigma^{2}}$$

where N is the total number of countries; $\sigma^{2}$ is the variance of factor in the study area; *h* = 1, 2, …, L is strata of variable. The value of the *q*-statistic ranged from 0 to 1. A *q*-statistic of 0 represents that the factor’s explanatory power is not significant, while a value of 1 indicates perfect explanatory power. To perform the analysis in the Geodetector, we reclassified factors into 5 levels using the natural break classification method (30). Spatial correlation analysis was performed using Geodetector software (http://geodetector.cn/).

The spatial lag model is a spatial regression model applied to quantify the effect of long-term stable factors, such as socioeconomic status, local economic development, geographic environment, and living conditions (31, 32). The equation for the calculation is as follows:


$$s_{i}=\rho W_{s_{i}}+X\beta +\emptyset$$

where $s_{i}$ is the dependent variable for a specific location. ρ is the spatial autoregressive coefficient of the lag term, which measures the extent to which the value of $s_{i}$ in a location is influenced by the values of $s_{i}$ in neighboring locations. W is the spatial adjacent matrix, reflecting the spatial trend of the response variables. X are all selected socioeconomic and environmental factors in this study. β is the spatial regression coefficient of the explanatory variables. ∅ is the error term of the spatial autocorrelation. Regression analyses, including OLS and Spatial lag model, were performed using the R (v4.3.2, https://www.r-project.org/).

## 3 Results

### 3.1 Spatial distribution and temporal trends of diabetes mortality

**Figure 1:**
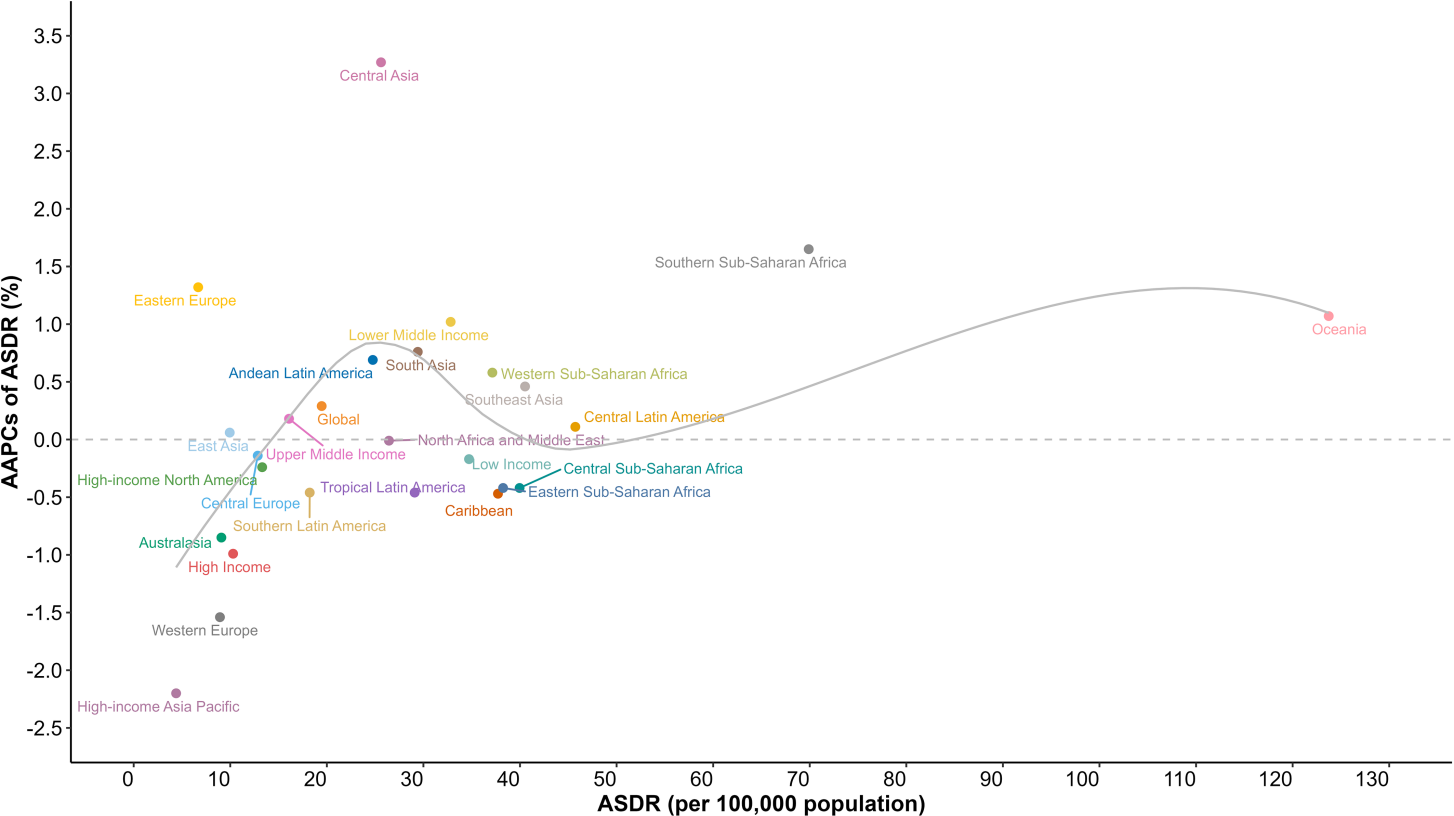
Age-standardized death rates of diabetes mellitus for GBD regions, and World Bank income levels by AAPC in 2019; the gray line represents expected values based on the AAPC and rates in GBD regions. AAPC, average annual percentage change; ASDR, age-standardized death rate; GBD, Global Burden of Disease Study.

Globally, the age-standardized mortality rate of diabetes increased from 17.92 (95% uncertainty interval [UI], 16.89 to 18.82) per 100,000 population in 1990 to 19.47 (18.08, 20.71) per 100,000 population in 2019, corresponding to an increase AAPC of 0.29% (95%CI, 0.27% to 0.31%) (**Figure 1**, **Supplementary Table S1**).

Regionally, diabetes mortality rates exhibited significant geographic variations across the GBD regions (**Figure 1**, **Supplementary Table S1**). In 2019, Oceania reported the highest diabetes mortality rate (123.74 [95%UI, 102.16 to 149.28] per 100,000), followed by Southern Sub-Saharan Africa (69.89 [95%UI, 64.51 to 75.17] per 100,000) and Central Latin America (45.73 [95%UI, 40.38 to 51.42] per 100,000). In contrast, the region with the lowest mortality rate was High-income Asia Pacific (4.39 [95%UI, 3.89 to 4.77] per 100,000), followed by Eastern Europe (6.68 [95%UI, 5.94 to 7.41] per 100,000) and West Europe (8.93 [95%UI, 8.01 to 9.46] per 100,000). Notably, the mortality rate in Oceania exceeded the global average by approximately sixfold. In addition, from 1990 to 2019, Oceania experienced a consistent increase in mortality rates, corresponding to an AAPC of 1.07% (95%CI, 1.03% to 1.11%).The highest increase in mortality rate was observed in Central Asia (-2.20% [95%CI, -2.27% to -2.14%]), while High-income Asia Pacific (3.27% [95%CI, 3.03% to 3.48%])experienced the most substantial decline in mortality rates.

When considering income levels, many disparities in diabetes mortality rates between countries emerge (**Figure 1**, **Supplementary Table S2**). In 2019, diabetes mortality rates ranked from low to high as follows: HICs (10.29 [9.43, 10.82] per 100,000), UMICs (16.08 [14.72, 17.33] per 100,000), LMICs (32.82 [29.97, 35.54] per 100,000), and LICs (34.72 [31.08, 38.7] per 100,000). This rank remained consistent over the past three decades. Notably, HICs (-0.99% [95%, -1.03% to -0.96%]) experienced a substantial decline in diabetes mortality and LICs (-0.17% [95%, -0.18% to -0.16%]) saw a slight decline, both exhibiting a continuous downward trend. Conversely, LMICs (1.02% [95%, 0.95% to 1.08%]) experienced a consistent and substantial increase in diabetes mortality, with the gap between LMICs mortality rate and that of LICs was steadily narrowing.

At the country level, the disparities in diabetes mortality exhibited significant magnitude (**Supplementary Table S1**). In 2019, the three countries with the highest mortality rates were Fiji (260.75 [212.99 to 313.42] per 100,000), Kiribati (206.61 [160.67 to 256.04] per 100,000), and the Federated States of Micronesia (171.59 [128.74 to 229.5] per 100,000), all located in Oceania. Notably, among Oceania countries, 17 countries had mortality rates three times higher than the global average, and 14 countries had rates five times higher. Among Latin America and the Caribbean, 13 countries reported mortality rates three times higher than the global average, and high mortality countries were concentrated in the Caribbean and vicinity. In Sub-Saharan African countries, only 7 countries reported mortality rates three times higher than the global average, whereas all of them showed an increasing trend in mortality. High mortality rate countries in Sub-Saharan Africa were all located in southern and central Africa. In contrast, the lowest mortality rates were observed in Japan (2.08 [1.8 to 2.25] per 100,000), Singapore (2.45 [2.13 to 2.7] per 100,000) and Belarus (2.52 [2.02 to 3.17] per 100,000).

### 3.2 Spatial autocorrelation of diabetes mortality

Using spatial autocorrelation analysis, the Moran’s *I* value for diabetes mortality rates from 1990 to 2019 ranged from 0.39 to 0.42, with all *Z*-score > 2.58 and *P*< 0.05, indicating consistent spatial clustering of diabetes mortality (**Table 2**). Countries tend to be neighboring countries with similar mortality rates. In addition, the results of the spatial correlation of diabetes mortality indicated that the distribution of diabetes is associated with potential geographic risk factors.

**Table 2:** Global Moran’s *I* of the age-standardized rate of diabetes mortality between 1990 and 2019.

Year	Moran’s *I*	*Z*-score	*P*-value	Spatial pattern
1990	0.420	23.309	<0.001	Clustering
1995	0.422	23.649	<0.001	Clustering
2000	0.403	22.706	<0.001	Clustering
2005	0.398	22.355	<0.001	Clustering
2010	0.392	22.031	<0.001	Clustering
2015	0.409	22.944	<0.001	Clustering
2019	0.422	23.682	<0.001	Clustering

**Figure 2:**
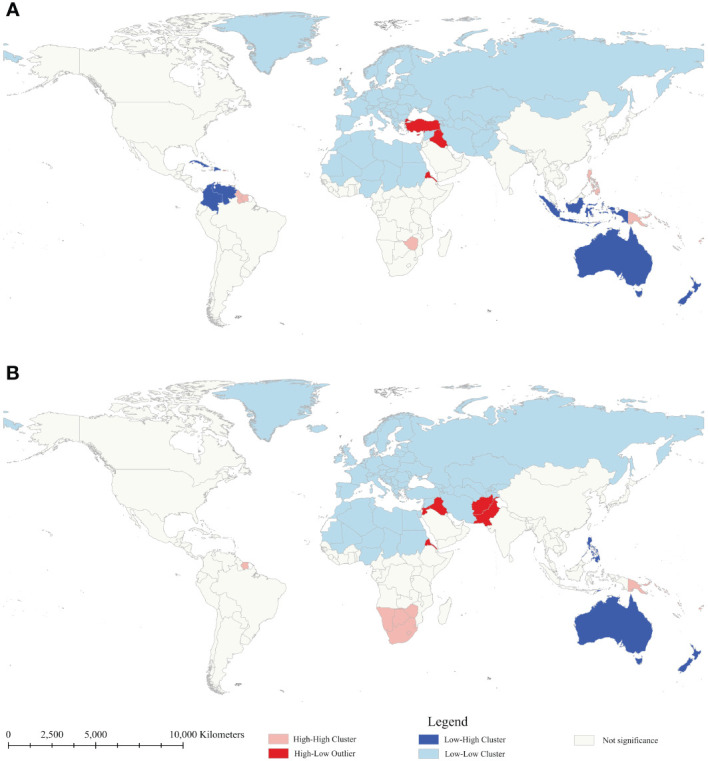
Clusters and outliers of age-standardized mortality rate of diabetes mellitus between 1990 **(A)** and 2019 **(B)**.

**Figure 3:**
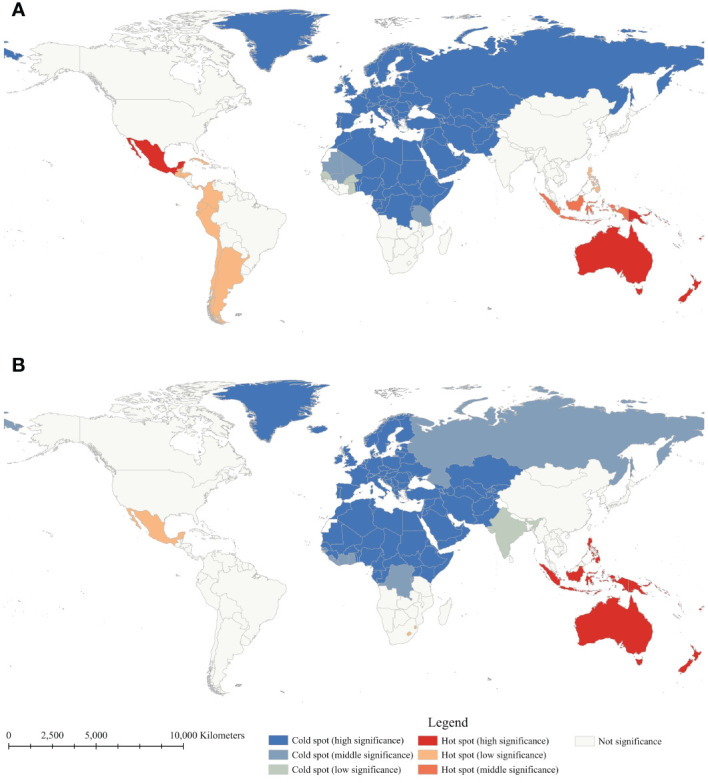
Getis-Ord *G_i_* ^*^ of age-standardized mortality rate of diabetes mellitus between 1990 **(A)** and 2019 **(B)**.

Anselin local Moran’s *I* and Getis-Ord *G_i_* ^*^ were employed to determine the local spatial autocorrelation of diabetes mortality rates between 1990 and 2019 (**Figures 2**, **3**). Throughout the study period, there was observed a relatively stable global distribution of clusters and outliers, as well as hot and cold spots. Through Anselin local Moran’s *I*, clusters of diabetes mortality rates were persistently distributed in countries within Oceania, and Southern Sub-Saharan Africa, indicating spatial clustering of mortality rates in these countries. In 2019, high-low outliers were observed in Afghanistan, Pakistan, Iraq, Jordan, Palestine, and Eritrea, presenting countries with high mortality rates surrounded by low rates. Conversely, low-high outliers were observed in Australia, New Zealand, the Philippines, and Timor-Leste, presenting countries with low mortality rates surrounded by high rates. Following Anselin local Moran’s *I*, Getis-Ord *G_i_* ^*^ was utilized to assess the significance of spatial clustering in diabetes mortality rates. The results reveal the essentially consistent distribution of cold and hot spots and clusters. Notably, hot spots in Oceania consistently maintained the highest level of significance, with those in Southeast Asia ranging from middle to high significance. Due to the significantly high mortality rates in Oceania countries, Australia and New Zealand, countries with low mortality rates, have also been considered hotspots. Hot spots in Latin America and the Caribbean countries exhibited a downward trend in statistical significance, indicating a mortality decline in these countries. Generally, stable spatial clustering with high significance was mostly found in island countries, such as countries in Oceania and the Caribbean. Furthermore, in comparison to Oceania countries, African countries, except for those in southern Africa, exhibited significantly lower mortality rates, explained the absence of persistent hot spots in Africa. However, with the rise in mortality rates in southern African countries, some countries in southern Africa have also shifted to hot spots.

### 3.3 Spatial heterogeneity analyses of diabetes mortality

The Geodetector was employed to explore the spatial correlation between diabetes mortality rates and risk factors (**Table 3**). The *q*-statistic values for 11 risk factors ranged from 0.055 to 0.373, with all *P*-value< 0.05, indicating the significant spatial correlation between mortality and these factors. Among these factors, universal health coverage exhibited the strongest correlation with mortality, closely followed by per capita health expenditure and human development index. In contrast, factors such as ambient air pollution, overweight in children, and high body-mass index exhibited relatively weaker correlations with mortality.

**Table 3:** The *q*-statistic of risk factors for diabetes mortality in 2019 by Geodetector.

Category	Risk factor	*q*-statistic	*P*-value
Health and Healthcare	Per capita health expenditure	0.339^**^	<0.001
	Universal health coverage	0.373^**^	<0.001
	Number of physicians	0.188^**^	<0.001
	High body-mass index	0.098^*^	0.011
	Overweight in children	0.070^*^	0.013
	Alcohol consumption	0.209^**^	<0.001
	Tobacco smoking prevalence	0.129^**^	<0.001
Environment	Household air pollution	0.187^**^	0.000
	Ambient air pollution	0.055^*^	0.043
Development and wellbeing	Urban population	0.113^**^	<0.001
	Human Development Index	0.299^**^	<0.001

Statistical significance level: *p< 0.05; **p< 0.01.

Through the OLS model, VIF results revealed that all 11 risk factors exhibited values lower than 5, indicating the absence of multicollinearity among these factors (**Supplementary Table S2**). The fit statistics of the OLS model presented *R* ^2^ and AIC values of 0.34 and 1995.20, respectively (**Supplementary Tables S3, S4**). Moran’s *I* of residual for the OLS model remained statistically significant, indicating the model failed to capture spatial structures in the data. Both the LM and Robust LM tests conducted for the spatial lag model yielded statistically significant results, thereby affirming the reliability of the model and avoiding multiple hypotheses. By incorporating spatial weight, and error terms into the model, the spatial lag model exhibited an improvement compared to the OLS model, with an elevated *R* ^2^ (0.39) and a reduced AIC (1993.24). Furthermore, Moran’s *I* of residual for the spatial lag model was not significant, indicating that these factors adequately addressed spatial effects on diabetes mortality rates.

After the spatial correlation, the spatial lag model was utilized to conduct spatial regression, quantifying the impact of risk factors on spatial heterogeneity of diabetes mortality (**Table 4**). Among these factors, seven exhibited significant spatial impact on spatial heterogeneity of diabetes mortality (*P*< 0.05). Notably, per capita health expenditure, number of physicians, alcohol consumption, and ambient air pollution exhibited negative correlations with mortality, while high body-mass index, tobacco smoking prevalence, and household air pollution displayed positive correlations with mortality. For instance, a 1000$ rise in health expenditure was associated with a 3.424% decrease in diabetes mortality. An increase in one physician per 1,000 people was associated with a 5.410% decrease in diabetes mortality. Conversely, a 1% rise in tobacco smoking prevalence was associated with a 0.498% increase in diabetes mortality. Similar patterns were observed for the remaining significant factors. On the other hand, while there was a significant spatial correlation with mortality rates, the spatial impact of universal health coverage, overweight in children, and urban population on mortality rates was not statistically significant (*P* > 0.05).

**Table 4:** The coefficient of risk factors for diabetes mortality in 2019 by Spatial Lag Model.

Category	Risk factor	Coefficient (95% CI)	*P*-value
Health and Healthcare	Per capita health expenditure	-3.424 (-8.556, -1.205)	0.039
	Universal health coverage	0.102 (-0.233, 0.610)	0.591
	Number of physicians	-5.410 (-11.332, -2.773)	0.005
	High body-mass index	1.252 (1.237, 2.377)	<0.001
	Overweight in children	-0.508 (-1.899, 0.221)	0.286
	Alcohol consumption	-0.910 (-2.978, -0.186)	0.148
	Tobacco smoking prevalence	0.498 (0.214, 1.125)	0.015
Environment	Household air pollution	0.271 (0.186, 0.600)	0.004
	Ambient air pollution	-0.457 (-1.218, -0.477)	0.008
Development and wellbeing	Urban population	-0.108 (-0.417, 0.094)	0.351
	Human Development Index	24.527 (-12.764, 55.471)	0.109

## 4 Discussion

Diabetes is a significant public health challenge globally. Gaining insights into the distribution and spatial determinants of diabetes mortality is pivotal for reducing mortality rates and narrowing the inequalities in diabetes deaths across countries. Employing spatial statistics, this study highlighted the distribution of high diabetes mortality, and quantified multiple spatial relationships between diabetes mortality and social and environmental factors. Through these, many overlooked or counterintuitive patterns in diabetes distribution and the relationship with risk factors have been revealed. The findings underscore the notable regional clustering of diabetes mortality rates and illuminate the multifaceted nature of the association with risk factors.

Deaths due to diabetes represent a widespread global health burden, and exhibit significant regional clustering. Over the past three decades, the global diabetes mortality rate has remained below 20 per 100,000. Diabetes mortality in countries within East Asia, Europe, and North America was consistently significantly lower than the global mortality rate. Conversely, in some regions, the mortality rate has been significantly higher than the global average. High diabetes mortality is primarily concentrated in countries with regions such as Oceania, Latin America and the Caribbean, and Sub-Saharan Africa, with most of these countries having mortality rates approximately twice as high as the global average. Among these countries, specifically countries in Oceania, the Caribbean, and Southern Africa, the mortality rates were even more than 3 to 12 times higher than the global average mortality rate. This indicates that diabetes mortality rates are significantly higher in many island countries, which is a conspicuous pattern of attention.

Compared to the significant differences in diabetes mortality rates observed when classified by geographic regions, the differences in mortality rates based on income levels are much smaller. The diabetes mortality rates in LICs and LMICs are approximately 2 to 3 times higher than in UMICs and HICs, and even less than twice the global mortality rate. Notably, in high diabetes mortality countries, there was also a significant disparity in income levels. In Sub-Saharan Africa, a region with generally high diabetes mortality rates, the majority of countries are LICs or LMICs, with mortality rates approximately twice the global average. In regions with exceptionally high mortality rates such as Oceania, the Caribbean, and Southern Africa, the majority of countries are UMICs or HICs. These reflect the complexity of diabetes mortality, with geographic location and income levels both potentially impacting diabetes mortality, requiring multifaceted analysis.

High diabetes mortality displays a conspicuous pattern of geographic clustering. Significantly high rates of diabetes mortality were concentrated in Oceania and the Caribbean, regions exclusively comprised of island countries. Notably, in Oceania, island countries such as Fiji, Kiribati, Nauru, and Micronesia consistently exhibited alarmingly high diabetes mortality, with rates 5 to 12 higher than the global average. This trend in island countries can be attributed to multifaceted factors, including geographical constraints, economic structure, and dietary practices. Previous studies reported that countries in the Pacific islands experienced great changes in social and economic structure (33). These changes have profoundly impacted the traditional economic frameworks of these nations, largely due to external influences. Substantial imported foods with high sugar substituted local traditional foods, such as fish and vegetables, which had dramatically increased sugar consumption among Pacific Islanders (34). This shift towards an unhealthy diet, coupled with a decline in physical activity, has led to a rapid escalation in obesity rates and diabetes prevalence, culminating in a stark rise in diabetes mortality. Similarly, these transitions have been observed in the Caribbean and adjacent countries (35, 36). For example, in Jamaica, an upper-middle-income country with a high prevalence of obesity and diabetes, economic shifts have been intricately associated with shifts in dietary habits (36). Cheaper foods, such as processed meat, sweetened beverages, and simple carbohydrates, contributed to a considerable increase in sugar consumption. The insufficient diagnosis and treatment of diabetes have not been effective in curbing prevalence and mortality (37). Furthermore, Southern Africa had a significantly high mortality concentrated, with rates even exceeding the global average by more than 3 to 5 times. Among these countries, those with limited land areas or located on islands, such as Eswatini, Lesotho, and Mauritius, reported the highest mortality rates (38). These finding suggest that island countries or countries with limited land areas particularly vulnerable when it comes to addressing diabetes in the face of external shocks. Geographical location and socioeconomic constraints hinder these countries from making timely adjustments to address diabetes, ultimately significantly increasing the health burden from diabetes.

The findings also exhibited the intricate spatial relationships between diabetes mortality and multifaceted risk factors, at the country level. The Geodetector results exhibited that there was a significant spatial correlation between diabetes mortality and all risk factors. The spatial regression results, however, displayed the intricate impact of risk factors on diabetes mortality.

Among these risk factors, several exhibited significant negative impacts on spatial heterogeneity of diabetes mortality, including per capita health expenditure, and number of physicians. Conversely, tobacco smoking prevalence, household air pollution, and high body-mass index displayed significant positive impacts on diabetes mortality. For these factors, our findings are consistent with previous studies at the individual level. As a chronic and complex disease, diabetes requires effective and long-term diagnosis and treatment, generally associated with adequate health expenditure and physicians (4, 6). Prioritizing countries with low per capita health expenditure and number of physicians would be highly beneficial for reducing the overall mortality rate. Notably, despite most countries within Oceania being UMICs or HICs, the per capita health expenditure and number of physicians in Oceania were both merely one-third of the world average in 2019, and slightly higher than those in Sub-Saharan Africa (39). Alongside rapid economic development, it is imperative to correspondingly enhance the quality of healthcare services, which is essential for mitigating the burden of diabetes mortality. Meanwhile, prolonged exposure to household air pollution is disadvantageous to people with diabetes and heightens the risk of diabetes mortality (40). In both Oceania and Sub-Saharan Africa, a substantial proportion of the population still relies on polluting fuels and technologies for cooking and heating (12). Promoting economic development and encouraging the replacement of polluted energy sources with clean energy sources is also beneficial for reducing diabetes-related mortality in these countries. Moreover, reducing tobacco smoking prevalence can further mitigate the risk of diabetes mortality at the country level (41). These findings suggest that these risk factors have similar impacts on diabetes mortality at the country level as at the individual level. Improving these risk factors on a global scale can effectively alleviate the burden of diabetes-related mortality.

However, the spatial regression results also revealed different outcomes compared to individual-level studies. In this analysis, alcohol consumption, and ambient air pollution were found to exert a significant negative impact on diabetes mortality. Previous studies at the individual level have demonstrated that long-term ambient air pollution exposure was associated with an increased risk of diabetes mortality (14). Nevertheless, ambient air pollution predominantly arises from industrial emissions and transportation (42). Regions with high ambient air pollution levels were primarily observed in South Asia, Central Asia, Northern Africa, and Western Asia, where most countries had persistently moderate diabetes mortality rates (43). Most countries with significantly high diabetes mortality rates, such as those in Oceania and Southern Africa, experienced ambient air pollution levels that were only about half to two-thirds of the global average ambient air pollution (21). Furthermore, household air pollution, which has a more direct impact on individuals, often surpasses ambient air pollution concentrations (42, 44). Meanwhile, a similar pattern emerged in the case of alcohol consumption. While alcohol consumption can be detrimental to the health of people with diabetes, countries with high alcohol consumption were primarily highly developed HICs, such as European countries, Australia, and New Zealand (45). In contrast, alcohol consumption in Oceania was merely half of the global average alcohol consumption, and that in Sub-Saharan Africa was slightly lower than the global average (21). Overall, despite improving ambient air pollution and reducing alcohol consumption being advantageous to people with diabetes, the impact of these factors was different at the country level. These factors were generally lower in countries with high diabetes mortality rates. Tailoring appropriate measures based on specific national circumstances can more effectively reduce the burden of diabetes mortality.

There are several limitations in this study. First, our study relied on the GBD 2019 data, which were constrained by limitations in primary data availability, discordant case definitions, and collinearity among covariates. Second, constrained by data availability, this study conducted spatial analysis at the country level. However, it is crucial to note that spatial heterogeneity also varied significantly even within countries with large areas, such as Canada, China, the United States, and Russia. Future studies would benefit from delving into sub-country levels to unravel the nuanced associations between diabetes burden and risk factors. Last, certain social indicators related to diabetes mortality were not considered as risk factors in this study due to data disparity and feasibility, such as healthcare services accessibility and educational levels. In future studies, the data involving more indicators will be collected and analyzed to strengthen the spatial relationship analysis.

## 5 Conclusion

The present study described comprehensive spatiotemporal dynamics of diabetes mortality and its spatial relationships with social, economic, and environmental risk factors from 1990 to 2019. The high diabetes mortality rates were primarily concentrated in countries with limited land areas or located on islands, such as countries in Oceania and the Caribbean. When considering income levels, LICs, and LMICs exhibited higher diabetes mortality in general, whereas significantly high rates of diabetes mortality were primarily observed in HICs or UMICs with rapid economic growth but relatively fragile. Spatial relationship analysis revealed that several risk factors exhibited an impact on diabetes mortality at the country level, consistent with findings at the individual level. These factors included per capita health expenditure, number of physicians, tobacco smoking prevalence, household air pollution, and high body-mass index. These findings offer a comprehensive depiction of the spatiotemporal distribution and seasonal variations in diabetes mortality across different geographic regions.

## Data availability statement

The original contributions presented in the study are included in the article/**Supplementary Material**. Further inquiries can be directed to the corresponding authors.

## Ethics statement

The studies involving humans were approved by the GBD 2019 https://vizhub.healthdata.org/gbd-results/. The studies were conducted by the local legislation and institutional requirements. Written informed consent for participation was not required from the participants or the participants’ legal guardians/next of kin by the national legislation and institutional requirements.

## Author contributions

ZX: Conceptualization, Data curation, Formal analysis, Investigation, Methodology, Resources, Validation, Visualization, Writing – original draft, Writing – review & editing. JF: Validation, Funding acquisition, Visualization, Writing – review & editing. SX: Investigation, Visualization, Writing – review & editing. YL: Investigation, Visualization, Writing – review & editing. YC: Investigation, Visualization, Writing – review & editing. JL: Funding acquisition, Project administration, Supervision, Writing – original draft, Writing – review & editing. YF: Conceptualization, Funding acquisition, Methodology, Project administration, Writing – original draft, Writing – review & editing.
