# Supplementary material for: Global trends and spatial drivers of diabetes mellitus mortality, 1990-2019: a systematic geographical analysis
Source: Front Endocrinol (Lausanne). 2024 Apr 12;15:1370489. doi: 10.3389/fendo.2024.1370489 (PMC11045957; doi:10.3389/fendo.2024.1370489)
Supplement: Supplementary file 1 [file Table_1.docx]

Supplementary Material

**Supplementary Table S1.** The original values of age-standardized mortality rate of diabetes in 2019, along with their average annual percent change (AAPC) from 1990 to 2019, by location.

| location | Income level | Age-standardized mortality rate per 100,000 (95% UI) | | AAPC (%) |
| --- | --- | --- | --- | --- |
|  |  | 1990 | 2019 |  |
| ***Global*** | - | 17.92 (16.89, 18.82) | 19.47 (18.08, 20.71) | 0.29 (0.27, 0.31) |
| ***World Bank income levels*** | | | | |
| High Income | - | 13.68 (12.85, 14.07) | 10.29 (9.43, 10.82) | -0.99 (-1.03, -0.96) |
| Low Income | - | 36.40 (32.38, 40.63) | 34.72 (31.08, 38.70) | -0.17 (-0.18, -0.16) |
| Lower Middle Income | - | 24.62 (22.30, 27.25) | 32.82 (29.97, 35.54) | 1.02 (0.95, 1.08) |
| Upper Middle Income | - | 15.40 (14.52, 16.32) | 16.08 (14.72, 17.33) | 0.18 (0.12, 0.24) |
| ***GBD regions*** | | | | |
| High-income North America | - | 14.36 (13.55, 14.80) | 13.31 (12.42, 13.79) | -0.24 (-0.29, -0.19) |
| Central Latin America | - | 44.85 (42.73, 46.11) | 45.73 (40.38, 51.42) | 0.11 (-0.07, 0.21) |
| Andean Latin America | - | 20.30 (18.45, 22.72) | 24.76 (20.72, 29.19) | 0.69 (0.54, 0.82) |
| Tropical Latin America | - | 33.50 (31.45, 34.90) | 29.09 (26.33, 30.66) | -0.46 (-0.52, -0.41) |
| Southern Latin America | - | 20.91 (19.87, 21.64) | 18.23 (16.91, 19.29) | -0.46 (-0.56, -0.38) |
| Caribbean | - | 43.83 (40.69, 46.81) | 37.72 (31.75, 44.62) | -0.47 (-0.57, -0.38) |
| Western Europe | - | 13.83 (12.92, 14.29) | 8.93 (8.01, 9.46) | -1.54 (-1.60, -1.49) |
| Central Europe | - | 13.32 (12.81, 13.68) | 12.84 (11.21, 14.64) | -0.14 (-0.22, -0.07) |
| Eastern Europe | - | 4.80 (4.64, 4.93) | 6.68 (5.94, 7.41) | 1.32 (0.67, 1.71) |
| North Africa and Middle East | - | 26.64 (24.16, 29.61) | 26.43 (23.49, 29.61) | -0.01 (-0.06, 0.04) |
| Central Sub-Saharan Africa | - | 45.03 (39.10, 51.31) | 39.96 (33.58, 48.26) | -0.42 (-0.46, -0.39) |
| Eastern Sub-Saharan Africa | - | 43.38 (38.25, 49.39) | 38.25 (34.06, 42.73) | -0.42 (-0.46, -0.40) |
| Western Sub-Saharan Africa | - | 31.49 (27.39, 36.02) | 37.14 (32.16, 41.87) | 0.58 (0.56, 0.59) |
| Southern Sub-Saharan Africa | - | 44.07 (39.01, 48.75) | 69.89 (64.51, 75.17) | 1.65 (1.47, 1.81) |
| Central Asia | - | 10.13 (9.79, 10.55) | 25.61 (23.27, 28.34) | 3.27 (3.03, 3.48) |
| East Asia | - | 9.76 (8.75, 10.91) | 9.94 (8.63, 11.31) | 0.06 (-0.02, 0.15) |
| High-income Asia Pacific | - | 8.31 (7.84, 8.65) | 4.39 (3.89, 4.77) | -2.20 (-2.27, -2.14) |
| South Asia | - | 23.35 (20.42, 27.27) | 29.41 (26.16, 33.06) | 0.76 (0.57, 0.95) |
| Southeast Asia | - | 35.68 (32.21, 38.91) | 40.52 (36.61, 44.43) | 0.46 (0.42, 0.49) |
| Australasia | - | 11.46 (10.70, 11.96) | 9.08 (8.12, 9.78) | -0.85 (-0.96, -0.76) |
| Oceania | - | 90.59 (76.23, 110.81) | 123.74 (102.16, 149.28) | 1.07 (1.03, 1.11) |
| ***GBD countries and territories*** | | | | |
| Afghanistan | Low | 30.93 (20.89, 45.05) | 42.31 (24.87, 61.67) | 1.10 (1.05, 1.13) |
| Albania | Upper-middle | 5.15 (4.67, 5.64) | 4.10 (3.07, 5.40) | -0.82 (-1.09, -0.63) |
| Algeria | Lower-middle | 18.47 (13.82, 25.46) | 19.19 (14.73, 24.63) | 0.13 (0.10, 0.17) |
| American Samoa | Upper-middle | 81.69 (72.37, 95.85) | 99.46 (85.96, 115.08) | 0.69 (0.62, 0.77) |
| Andorra | High | 7.94 (6.06, 10.60) | 6.09 (4.66, 7.69) | -0.91 (-0.93, -0.88) |
| Angola | Lower-middle | 43.11 (34.53, 52.37) | 42.96 (35.86, 52.37) | -0.04 (-0.10, 0.01) |
| Antigua and Barbuda | High | 61.04 (55.79, 65.75) | 61.23 (53.36, 69.96) | -0.00 (-0.11, 0.11) |
| Argentina | Upper-middle | 22.71 (21.48, 23.64) | 19.62 (18.24, 21.00) | -0.53 (-0.64, -0.43) |
| Armenia | Upper-middle | 19.06 (18.04, 20.04) | 29.96 (24.96, 34.89) | 1.34 (0.82, 1.57) |
| Australia | High | 11.56 (10.73, 12.14) | 9.24 (8.22, 10.02) | -0.82 (-0.92, -0.73) |
| Austria | High | 12.79 (11.91, 13.38) | 11.36 (10.18, 12.27) | -0.49 (-0.60, -0.38) |
| Azerbaijan | Upper-middle | 12.08 (11.04, 13.38) | 24.82 (20.38, 29.67) | 2.60 (2.51, 2.69) |
| Bahamas | High | 51.66 (47.07, 56.44) | 40.37 (33.79, 48.71) | -0.88 (-1.05, -0.72) |
| Bahrain | High | 79.39 (67.66, 93.09) | 128.02 (103.41, 155.93) | 1.55 (1.28, 1.77) |
| Bangladesh | Lower-middle | 29.09 (24.78, 33.41) | 30.77 (24.58, 37.24) | 0.27 (0.06, 0.53) |
| Barbados | High | 72.11 (67.10, 76.51) | 63.53 (53.31, 73.82) | -0.50 (-0.65, -0.38) |
| Belarus | Upper-middle | 4.89 (4.66, 5.12) | 2.52 (2.02, 3.17) | -2.31 (-2.96, -1.80) |
| Belgium | High | 11.78 (10.85, 12.42) | 6.45 (5.77, 6.98) | -1.97 (-2.10, -1.82) |
| Belize | Upper-middle | 45.56 (41.28, 49.45) | 58.64 (51.39, 66.61) | 0.84 (0.65, 1.05) |
| Benin | Lower-middle | 28.37 (24.12, 33.26) | 36.01 (28.50, 45.67) | 0.82 (0.80, 0.83) |
| Bermuda | High | 33.50 (30.79, 36.21) | 17.95 (15.10, 21.68) | -2.13 (-2.23, -2.02) |
| Bhutan | Lower-middle | 19.91 (14.80, 25.79) | 31.65 (24.31, 40.49) | 1.59 (1.57, 1.62) |
| Bosnia and Herzegovina | Upper-middle | 16.43 (15.19, 17.83) | 38.49 (29.48, 47.36) | 3.05 (2.89, 3.16) |
| Botswana | Upper-middle | 61.49 (48.33, 79.54) | 92.48 (70.93, 118.79) | 1.43 (1.39, 1.46) |
| Brazil | Upper-middle | 33.76 (31.72, 35.21) | 28.70 (26.01, 30.28) | -0.54 (-0.60, -0.48) |
| Brunei Darussalam | High | 114.75 (99.07, 134.43) | 75.02 (66.98, 84.43) | -1.50 (-1.61, -1.38) |
| Bulgaria | Upper-middle | 17.06 (16.10, 18.00) | 16.00 (12.96, 19.61) | -0.17 (-0.30, -0.06) |
| Burkina Faso | Low | 36.62 (30.55, 43.25) | 36.82 (30.45, 43.61) | 0.06 (0.01, 0.11) |
| Burundi | Low | 47.18 (37.34, 62.07) | 37.48 (29.94, 46.77) | -0.80 (-0.86, -0.75) |
| Cabo Verde | Lower-middle | 9.57 (8.29, 12.76) | 32.47 (27.31, 37.93) | 4.39 (4.22, 4.61) |
| Cambodia | Lower-middle | 34.59 (28.10, 41.67) | 33.96 (26.87, 40.60) | -0.07 (-0.08, -0.05) |
| Cameroon | Lower-middle | 37.59 (29.87, 47.09) | 51.00 (40.10, 64.78) | 1.04 (1.00, 1.08) |
| Canada | High | 13.52 (12.46, 14.20) | 10.11 (9.03, 10.95) | -0.97 (-1.06, -0.89) |
| Central African Republic | Low | 51.18 (41.79, 60.85) | 51.51 (40.04, 65.68) | 0.00 (-0.03, 0.03) |
| Chad | Low | 25.34 (20.19, 30.98) | 34.63 (27.24, 43.29) | 1.10 (1.07, 1.13) |
| Chile | High | 16.21 (15.41, 16.90) | 15.95 (14.36, 17.26) | -0.05 (-0.13, 0.02) |
| China | Upper-middle | 9.20 (8.18, 10.39) | 9.44 (8.10, 10.82) | 0.10 (0.02, 0.18) |
| Colombia | Upper-middle | 20.51 (19.16, 21.48) | 13.11 (10.34, 16.56) | -1.60 (-1.89, -1.40) |
| Comoros | Lower-middle | 39.29 (26.98, 48.68) | 36.91 (30.74, 45.61) | -0.16 (-0.25, -0.05) |
| Congo | Lower-middle | 59.86 (49.33, 72.01) | 53.42 (43.69, 64.72) | -0.40 (-0.43, -0.35) |
| Cook Islands | High | 123.64 (107.60, 144.27) | 111.71 (93.84, 132.04) | -0.34 (-0.39, -0.29) |
| Costa Rica | Upper-middle | 15.65 (14.45, 16.72) | 11.30 (8.84, 14.19) | -0.77 (-1.22, -0.39) |
| Croatia | High | 13.01 (12.14, 13.91) | 11.92 (9.57, 14.54) | -0.19 (-0.39, 0.02) |
| Cuba | Upper-middle | 22.45 (21.11, 23.41) | 10.39 (8.50, 12.61) | -2.54 (-2.80, -2.33) |
| Cyprus | High | 65.62 (51.38, 76.34) | 27.51 (22.45, 31.55) | -3.04 (-3.11, -2.95) |
| Czechia | High | 13.91 (13.18, 14.51) | 15.47 (12.70, 18.40) | 0.55 (0.15, 0.77) |
| Democratic People's Republic of Korea | Low | 14.37 (11.35, 18.21) | 13.10 (10.79, 16.13) | -0.32 (-0.33, -0.31) |
| Democratic Republic of the Congo | Low | 43.42 (36.56, 50.98) | 36.32 (29.54, 44.52) | -0.63 (-0.67, -0.58) |
| Denmark | High | 10.92 (10.37, 11.35) | 11.76 (10.62, 12.79) | 0.24 (0.16, 0.32) |
| Djibouti | Lower-middle | 35.15 (27.30, 44.82) | 43.67 (35.17, 55.88) | 0.74 (0.69, 0.78) |
| Dominica | Upper-middle | 69.13 (61.94, 76.59) | 63.34 (52.44, 75.70) | -0.37 (-0.43, -0.30) |
| Dominican Republic | Upper-middle | 23.13 (20.45, 26.07) | 32.59 (24.97, 41.96) | 1.20 (1.00, 1.36) |
| Ecuador | Upper-middle | 22.75 (21.29, 24.85) | 35.21 (28.37, 44.11) | 1.61 (1.35, 1.81) |
| Egypt | Lower-middle | 25.95 (24.10, 28.08) | 35.47 (27.46, 45.93) | 1.25 (0.96, 1.36) |
| El Salvador | Lower-middle | 18.44 (16.95, 20.82) | 37.54 (28.54, 48.09) | 2.64 (2.38, 2.83) |
| Equatorial Guinea | Upper-middle | 45.50 (34.43, 56.39) | 55.87 (41.45, 74.48) | 0.70 (0.65, 0.75) |
| Eritrea | Low | 39.44 (28.37, 55.55) | 46.91 (35.77, 61.09) | 0.59 (0.55, 0.62) |
| Estonia | High | 4.61 (4.34, 4.89) | 5.46 (4.32, 6.85) | 0.48 (0.18, 0.78) |
| Eswatini | Lower-middle | 85.36 (67.06, 103.43) | 136.60 (98.77, 179.41) | 1.57 (1.50, 1.65) |
| Ethiopia | Low | 57.91 (47.80, 73.10) | 36.00 (31.01, 41.43) | -1.64 (-1.69, -1.58) |
| Fiji | Upper-middle | 162.61 (133.55, 208.68) | 260.75 (212.99, 313.42) | 1.43 (1.07, 1.77) |
| Finland | High | 8.36 (7.78, 8.79) | 4.00 (3.65, 4.33) | -2.56 (-2.64, -2.49) |
| France | High | 8.51 (7.80, 9.01) | 7.58 (6.69, 8.27) | -0.40 (-0.50, -0.32) |
| Gabon | Upper-middle | 63.14 (48.62, 84.27) | 67.18 (52.59, 81.76) | 0.26 (0.21, 0.31) |
| Gambia | Low | 25.90 (20.56, 31.98) | 38.72 (29.11, 48.52) | 1.43 (1.28, 1.58) |
| Georgia | Upper-middle | 11.33 (10.46, 12.20) | 22.77 (19.10, 26.42) | 2.47 (2.32, 2.62) |
| Germany | High | 16.43 (15.20, 17.31) | 10.11 (9.10, 10.91) | -1.70 (-1.75, -1.63) |
| Ghana | Lower-middle | 30.24 (25.19, 36.03) | 44.12 (36.76, 53.01) | 1.31 (1.26, 1.35) |
| Greece | High | 7.65 (7.12, 8.06) | 5.11 (4.64, 5.50) | -1.53 (-1.66, -1.42) |
| Greenland | High | 16.21 (14.06, 18.69) | 9.31 (7.45, 11.18) | -1.83 (-1.97, -1.67) |
| Grenada | Upper-middle | 71.44 (64.76, 78.19) | 73.87 (66.95, 80.75) | 0.16 (0.06, 0.26) |
| Guam | High | 40.34 (34.08, 46.11) | 24.27 (20.33, 29.31) | -1.79 (-1.97, -1.65) |
| Guatemala | Upper-middle | 17.53 (15.66, 19.36) | 62.45 (50.14, 76.99) | 4.45 (4.16, 4.67) |
| Guinea | Low | 29.96 (24.25, 35.75) | 39.70 (31.02, 48.29) | 0.99 (0.97, 1.02) |
| Guinea-Bissau | Low | 43.37 (34.12, 55.72) | 51.04 (39.04, 63.92) | 0.57 (0.55, 0.59) |
| Guyana | Upper-middle | 85.13 (75.60, 94.46) | 96.97 (77.78, 119.68) | 0.54 (0.31, 0.77) |
| Haiti | Low | 84.83 (66.57, 105.82) | 66.31 (47.14, 93.31) | -0.85 (-0.87, -0.83) |
| Honduras | Lower-middle | 12.78 (10.24, 14.81) | 19.57 (15.93, 24.28) | 1.50 (1.39, 1.61) |
| Hungary | High | 12.72 (12.14, 13.26) | 12.32 (10.26, 14.72) | -0.01 (-0.26, 0.21) |
| Iceland | High | 5.29 (4.80, 5.77) | 4.22 (3.63, 4.78) | -0.69 (-0.78, -0.60) |
| India | Lower-middle | 22.30 (19.19, 26.33) | 27.35 (23.88, 31.58) | 0.65 (0.36, 1.00) |
| Indonesia | Upper-middle | 34.26 (30.66, 38.51) | 51.43 (43.87, 57.62) | 1.42 (1.40, 1.45) |
| Iraq | Upper-middle | 50.40 (40.34, 60.20) | 46.51 (37.84, 55.34) | -0.27 (-0.31, -0.24) |
| Ireland | High | 10.64 (9.99, 11.17) | 6.43 (5.65, 7.04) | -1.79 (-1.92, -1.66) |
| Israel | High | 17.62 (16.31, 18.51) | 21.11 (18.65, 22.79) | 0.50 (0.36, 0.62) |
| Italy | High | 19.55 (18.37, 20.25) | 12.28 (10.69, 13.07) | -1.60 (-1.75, -1.52) |
| Jamaica | Upper-middle | 64.05 (59.95, 67.35) | 77.41 (63.08, 94.47) | 0.87 (0.59, 1.16) |
| Japan | High | 6.41 (5.97, 6.65) | 2.08 (1.80, 2.25) | -3.83 (-3.89, -3.78) |
| Jordan | Upper-middle | 65.44 (55.03, 76.79) | 41.05 (34.87, 48.43) | -1.68 (-1.84, -1.51) |
| Kazakhstan | Upper-middle | 7.73 (7.31, 8.12) | 16.25 (14.15, 18.50) | 2.52 (2.16, 2.76) |
| Kenya | Lower-middle | 28.51 (25.47, 32.46) | 33.30 (28.26, 38.81) | 0.52 (0.48, 0.55) |
| Kiribati | Lower-middle | 149.51 (122.68, 179.72) | 206.61 (160.67, 256.04) | 1.12 (1.10, 1.15) |
| Kuwait | High | 29.44 (26.14, 32.91) | 18.75 (15.51, 22.50) | -1.64 (-2.09, -1.13) |
| Kyrgyzstan | Lower-middle | 6.64 (6.21, 7.10) | 7.02 (6.10, 8.01) | 0.27 (0.11, 0.47) |
| Lao People's Democratic Republic | Lower-middle | 41.80 (31.84, 54.07) | 39.72 (31.21, 49.53) | -0.17 (-0.20, -0.15) |
| Latvia | High | 5.37 (5.10, 5.68) | 8.31 (6.96, 9.92) | 1.88 (0.94, 2.25) |
| Lebanon | Upper-middle | 21.95 (19.05, 26.26) | 16.66 (11.89, 21.28) | -0.95 (-0.99, -0.91) |
| Lesotho | Lower-middle | 52.06 (42.91, 62.19) | 113.31 (84.33, 145.99) | 2.73 (2.67, 2.78) |
| Liberia | Low | 31.62 (26.72, 37.24) | 36.16 (27.37, 47.00) | 0.45 (0.36, 0.49) |
| Libya | Upper-middle | 15.60 (11.59, 19.67) | 19.49 (14.23, 25.69) | 0.74 (0.62, 0.85) |
| Lithuania | High | 4.52 (4.28, 4.78) | 4.32 (3.55, 5.27) | -0.00 (-0.31, 0.18) |
| Luxembourg | High | 11.05 (10.12, 11.88) | 6.15 (5.26, 7.09) | -2.03 (-2.19, -1.92) |
| Madagascar | Low | 32.08 (26.58, 38.33) | 31.40 (24.39, 40.09) | -0.02 (-0.13, 0.09) |
| Malawi | Low | 42.22 (36.02, 49.00) | 38.99 (32.81, 45.89) | -0.27 (-0.33, -0.23) |
| Malaysia | Upper-middle | 30.51 (27.25, 33.42) | 15.34 (12.16, 20.06) | -2.18 (-2.57, -1.81) |
| Maldives | Upper-middle | 34.72 (28.60, 43.73) | 20.07 (16.53, 23.93) | -1.96 (-2.07, -1.84) |
| Mali | Low | 30.94 (25.69, 36.73) | 36.28 (29.05, 43.85) | 0.56 (0.53, 0.59) |
| Malta | High | 24.23 (22.31, 26.04) | 12.37 (10.65, 13.96) | -2.27 (-2.37, -2.17) |
| Marshall Islands | Upper-middle | 85.41 (71.22, 102.33) | 115.35 (88.83, 148.89) | 1.01 (0.89, 1.11) |
| Mauritania | Lower-middle | 35.85 (29.98, 42.28) | 34.22 (26.73, 43.19) | -0.14 (-0.21, -0.09) |
| Mauritius | High | 49.05 (46.52, 51.75) | 112.09 (92.98, 135.12) | 2.81 (2.65, 2.94) |
| Mexico | Upper-middle | 65.85 (62.76, 67.85) | 65.41 (56.65, 74.40) | 0.08 (-0.06, 0.24) |
| Monaco | High | 3.57 (2.87, 4.35) | 3.44 (2.78, 4.09) | -0.13 (-0.14, -0.11) |
| Mongolia | Lower-middle | 5.36 (4.45, 6.46) | 5.80 (4.55, 7.33) | 0.26 (0.19, 0.33) |
| Montenegro | Upper-middle | 13.72 (11.88, 15.38) | 15.66 (13.12, 18.68) | 0.51 (0.39, 0.61) |
| Morocco | Lower-middle | 14.61 (11.67, 21.10) | 23.75 (18.18, 30.25) | 1.66 (1.60, 1.72) |
| Mozambique | Low | 38.70 (32.51, 45.33) | 47.86 (38.69, 59.62) | 0.75 (0.72, 0.78) |
| Myanmar | Lower-middle | 59.17 (46.34, 73.52) | 53.74 (46.73, 62.11) | -0.34 (-0.36, -0.32) |
| Namibia | Upper-middle | 58.78 (49.18, 68.30) | 63.23 (50.17, 79.41) | 0.26 (0.21, 0.30) |
| Nauru | High | 115.33 (92.46, 147.09) | 165.05 (128.93, 229.64) | 1.25 (1.22, 1.27) |
| Nepal | Lower-middle | 12.05 (9.63, 14.75) | 19.57 (15.50, 23.58) | 1.69 (1.66, 1.72) |
| Netherlands | High | 18.12 (16.63, 19.07) | 9.08 (8.16, 9.89) | -2.37 (-2.43, -2.31) |
| New Zealand | High | 11.05 (10.31, 11.69) | 8.16 (7.40, 8.79) | -0.99 (-1.13, -0.88) |
| Nicaragua | Lower-middle | 29.33 (26.83, 32.44) | 47.55 (40.23, 55.08) | 1.53 (1.27, 1.79) |
| Niger | Low | 26.73 (21.19, 32.84) | 32.11 (25.25, 39.37) | 0.67 (0.63, 0.70) |
| Nigeria | Lower-middle | 31.76 (26.38, 38.27) | 34.22 (27.69, 41.61) | 0.26 (0.24, 0.28) |
| Niue | High | 90.13 (73.67, 112.70) | 123.37 (96.42, 163.75) | 1.11 (1.07, 1.14) |
| North Macedonia | Upper-middle | 21.83 (19.45, 24.10) | 35.63 (28.62, 43.42) | 1.87 (1.77, 1.96) |
| Northern Mariana Islands | High | 51.36 (43.84, 61.59) | 59.51 (50.89, 68.98) | 0.39 (0.23, 0.56) |
| Norway | High | 7.78 (7.22, 8.09) | 6.23 (5.58, 6.67) | -0.79 (-0.86, -0.73) |
| Oman | High | 49.04 (37.75, 62.34) | 61.54 (53.34, 70.19) | 0.77 (0.65, 0.94) |
| Pakistan | Lower-middle | 24.49 (16.52, 29.76) | 51.26 (40.11, 62.72) | 2.60 (2.57, 2.62) |
| Palau | Upper-middle | 80.57 (65.21, 100.56) | 120.54 (93.17, 149.87) | 1.41 (1.39, 1.42) |
| Palestine | Lower-middle | 56.06 (44.68, 69.34) | 70.33 (60.83, 80.48) | 0.71 (0.59, 0.83) |
| Panama | High | 19.19 (17.50, 20.56) | 29.14 (22.84, 36.43) | 1.49 (1.27, 1.63) |
| Papua New Guinea | Lower-middle | 79.88 (63.15, 100.69) | 106.80 (84.67, 134.30) | 1.00 (0.98, 1.02) |
| Paraguay | Upper-middle | 23.85 (20.85, 26.79) | 46.32 (35.99, 58.47) | 2.44 (2.29, 2.59) |
| Peru | Upper-middle | 13.91 (12.07, 16.10) | 14.91 (11.20, 19.61) | 0.37 (0.12, 0.58) |
| Philippines | Lower-middle | 38.84 (35.02, 42.68) | 38.82 (32.64, 46.18) | 0.04 (-0.12, 0.16) |
| Poland | High | 13.92 (13.35, 14.31) | 11.26 (9.55, 13.18) | -0.73 (-0.98, -0.61) |
| Portugal | High | 20.59 (19.42, 21.56) | 14.74 (12.96, 16.04) | -1.16 (-1.28, -1.06) |
| Puerto Rico | High | 48.09 (44.87, 50.44) | 43.84 (34.40, 55.41) | -0.67 (-0.94, -0.47) |
| Qatar | High | 112.99 (96.00, 133.08) | 123.22 (99.88, 152.91) | 0.35 (0.10, 0.61) |
| Republic of Korea | High | 18.30 (17.14, 24.87) | 15.02 (13.00, 16.98) | -0.74 (-0.86, -0.59) |
| Republic of Moldova | Lower-middle | 7.28 (6.96, 7.64) | 4.93 (4.25, 5.67) | -1.12 (-1.74, -0.73) |
| Romania | High | 7.45 (7.15, 7.76) | 6.35 (5.25, 7.48) | -0.56 (-0.69, -0.45) |
| Bolivia (Plurinational State of) | Lower-middle | 40.92 (32.90, 50.61) | 46.45 (36.48, 58.49) | 0.44 (0.41, 0.47) |
| Côte d'Ivoire | Lower-middle | 34.36 (28.80, 40.09) | 40.15 (32.12, 49.13) | 0.54 (0.51, 0.58) |
| Iran (Islamic Republic of) | Upper-middle | 13.60 (11.80, 15.64) | 22.72 (19.06, 24.54) | 1.74 (1.66, 1.82) |
| Micronesia (Federated States of) | Lower-middle | 97.36 (77.40, 120.74) | 171.59 (128.74, 229.50) | 1.97 (1.95, 1.99) |
| Russian Federation | Upper-middle | 4.88 (4.72, 5.04) | 7.96 (6.95, 9.04) | 1.96 (1.19, 2.46) |
| Rwanda | Low | 52.30 (41.18, 66.55) | 38.28 (29.44, 49.59) | -1.11 (-1.16, -1.06) |
| Saint Kitts and Nevis | High | 74.68 (68.74, 81.05) | 56.26 (48.80, 65.19) | -0.98 (-1.17, -0.81) |
| Saint Lucia | Upper-middle | 87.35 (80.75, 92.93) | 63.12 (53.75, 73.78) | -1.00 (-1.20, -0.87) |
| Saint Vincent and the Grenadines | Upper-middle | 90.11 (82.55, 96.89) | 83.64 (73.91, 95.26) | -0.29 (-0.39, -0.19) |
| Samoa | Upper-middle | 73.73 (59.54, 93.05) | 90.73 (73.77, 113.06) | 0.71 (0.69, 0.73) |
| San Marino | High | 7.65 (6.43, 9.08) | 6.21 (4.21, 8.62) | -0.71 (-0.74, -0.68) |
| Sao Tome and Principe | Lower-middle | 12.83 (10.77, 14.83) | 16.54 (13.40, 19.54) | 0.90 (0.86, 0.95) |
| Saudi Arabia | High | 26.47 (20.25, 34.10) | 20.43 (16.76, 24.74) | -0.93 (-0.99, -0.86) |
| Senegal | Lower-middle | 30.67 (24.78, 36.94) | 39.61 (30.98, 49.00) | 0.86 (0.78, 0.94) |
| Serbia | Upper-middle | 21.36 (17.90, 23.93) | 22.75 (18.70, 27.57) | 0.27 (0.07, 0.50) |
| Seychelles | High | 17.18 (15.35, 19.22) | 26.43 (23.01, 29.85) | 1.52 (1.44, 1.58) |
| Sierra Leone | Low | 25.78 (21.04, 30.75) | 34.35 (26.78, 43.22) | 1.00 (0.97, 1.03) |
| Singapore | High | 18.63 (17.38, 19.60) | 2.45 (2.13, 2.70) | -6.92 (-7.12, -6.72) |
| Slovakia | High | 14.07 (12.95, 15.23) | 8.97 (7.08, 11.13) | -1.55 (-1.70, -1.40) |
| Slovenia | High | 11.56 (9.20, 14.24) | 7.34 (5.78, 9.33) | -1.56 (-1.91, -1.24) |
| Solomon Islands | Lower-middle | 79.74 (58.66, 112.98) | 136.51 (113.05, 166.37) | 1.84 (1.77, 1.92) |
| Somalia | Low | 45.69 (35.49, 58.81) | 45.10 (35.09, 56.98) | -0.03 (-0.06, -0.00) |
| South Africa | Upper-middle | 43.55 (37.10, 49.20) | 69.00 (63.35, 74.42) | 1.65 (1.32, 1.99) |
| South Sudan | Low | 36.75 (29.21, 46.19) | 35.28 (26.70, 45.02) | -0.16 (-0.18, -0.14) |
| Spain | High | 17.68 (16.05, 18.74) | 8.15 (6.99, 8.92) | -2.66 (-2.78, -2.58) |
| Sri Lanka | Lower-middle | 27.28 (24.41, 30.26) | 59.28 (44.54, 75.94) | 2.74 (2.58, 2.87) |
| Sudan | Low | 12.93 (9.72, 18.03) | 16.78 (11.40, 23.24) | 0.90 (0.88, 0.92) |
| Suriname | Upper-middle | 38.01 (34.64, 41.25) | 46.04 (38.66, 54.62) | 0.71 (0.56, 0.87) |
| Sweden | High | 9.38 (8.62, 9.98) | 8.48 (7.57, 9.13) | -0.36 (-0.41, -0.30) |
| Switzerland | High | 14.09 (12.84, 14.85) | 6.59 (5.68, 7.21) | -2.61 (-2.68, -2.54) |
| Syrian Arab Republic | Low | 21.87 (17.27, 26.60) | 18.21 (14.11, 23.44) | -0.73 (-0.83, -0.61) |
| Tajikistan | Low | 12.91 (11.93, 13.97) | 33.74 (26.97, 41.39) | 3.35 (3.16, 3.49) |
| Thailand | Upper-middle | 26.80 (22.96, 31.13) | 19.52 (14.61, 25.34) | -0.97 (-1.12, -0.83) |
| Timor-Leste | Lower-middle | 24.13 (19.02, 29.90) | 26.09 (20.19, 32.41) | 0.29 (0.23, 0.35) |
| Togo | Low | 27.80 (22.85, 33.44) | 34.52 (26.73, 44.44) | 0.77 (0.74, 0.79) |
| Tokelau | High | 77.64 (62.20, 95.70) | 97.87 (76.10, 124.87) | 0.79 (0.77, 0.80) |
| Tonga | Upper-middle | 87.73 (73.54, 102.83) | 105.94 (83.97, 130.88) | 0.70 (0.64, 0.76) |
| Trinidad and Tobago | High | 125.75 (119.80, 131.42) | 100.27 (77.06, 127.22) | -0.71 (-1.18, -0.28) |
| Tunisia | Lower-middle | 12.02 (9.70, 16.76) | 15.68 (11.33, 21.09) | 0.89 (0.84, 0.94) |
| Turkey | Upper-middle | 42.39 (36.26, 49.16) | 23.78 (19.11, 28.81) | -1.96 (-2.05, -1.87) |
| Turkmenistan | Upper-middle | 10.98 (10.39, 11.55) | 18.41 (14.79, 23.11) | 2.10 (1.86, 2.32) |
| Tuvalu | Upper-middle | 91.22 (74.71, 110.85) | 117.72 (89.73, 157.98) | 0.89 (0.88, 0.91) |
| Uganda | Low | 39.35 (30.76, 51.40) | 41.47 (32.47, 52.22) | 0.16 (0.13, 0.19) |
| Ukraine | Lower-middle | 4.45 (4.27, 4.61) | 4.05 (3.48, 4.72) | -0.05 (-0.54, 0.33) |
| United Arab Emirates | High | 78.65 (60.82, 97.01) | 57.16 (42.75, 72.93) | -1.16 (-1.39, -0.98) |
| United Kingdom | High | 8.44 (7.92, 8.71) | 4.62 (4.21, 4.85) | -2.12 (-2.19, -2.06) |
| United Republic of Tanzania | Lower-middle | 37.19 (31.78, 42.79) | 38.46 (31.47, 45.93) | 0.13 (0.09, 0.16) |
| United States of America | High | 14.49 (13.69, 14.94) | 13.69 (12.83, 14.17) | -0.17 (-0.22, -0.12) |
| United States Virgin Islands | High | 43.53 (37.14, 49.68) | 44.74 (38.50, 51.05) | 0.13 (0.02, 0.25) |
| Uruguay | High | 17.63 (16.56, 18.47) | 15.25 (13.95, 16.40) | -0.59 (-0.67, -0.51) |
| Uzbekistan | Lower-middle | 9.85 (9.32, 10.35) | 38.59 (32.67, 44.68) | 4.83 (4.52, 5.05) |
| Vanuatu | Lower-middle | 45.81 (33.60, 65.05) | 79.60 (59.88, 105.68) | 1.88 (1.83, 1.94) |
| Venezuela (Bolivarian Republic of) | Upper-middle | 33.71 (31.71, 35.35) | 38.55 (29.92, 49.16) | 0.18 (-0.04, 0.31) |
| Viet Nam | Lower-middle | 33.36 (26.51, 40.29) | 36.39 (29.31, 43.76) | 0.30 (0.29, 0.31) |
| Yemen | Low | 13.01 (9.33, 18.81) | 15.32 (11.05, 21.32) | 0.57 (0.55, 0.59) |
| Zambia | Lower-middle | 44.49 (37.84, 51.73) | 44.41 (36.77, 53.35) | -0.01 (-0.06, 0.02) |
| Zimbabwe | Lower-middle | 35.94 (31.27, 40.87) | 56.95 (44.90, 70.16) | 1.62 (1.54, 1.67) |

**Supplementary Table S2.** Variance inflation factors (VIF) for risk factors on age-standardized death rates of diabetes in 2019.

| Risk factor | VIF |
| --- | --- |
| Per capita health expenditure | 2.57 |
| Universal health coverage | 3.83 |
| Number of physicians | 2.71 |
| High body-mass index | 1.99 |
| Overweight in children | 1.34 |
| Alcohol consumption | 1.83 |
| Tobacco smoking prevalence | 1.43 |
| Household air pollution | 3.22 |
| Ambient air pollution | 1.49 |
| Urban population | 1.86 |
| Human Development Index | 3.42 |

**Supplementary Table S3.** The coefficient of risk factors for diabetes mortality by ordinary least squares (OLS).

| Risk factor | Estimate | Std. Error | t-value | *P*-value |
| --- | --- | --- | --- | --- |
| Per capita health expenditure | -4.880** | 0.002 | -2.619 | 0.010 |
| Universal health coverage | 0.189 | 0.214 | 0.884 | 0.378 |
| Number of physicians | -7.053** | 2.170 | -3.250 | 0.001 |
| High body-mass index | 1.807** | 0.289 | 6.248 | <0.001 |
| Overweight in children | -0.839 | 0.537 | -1.562 | 0.120 |
| Alcohol consumption | -1.582* | 0.708 | -2.235 | 0.027 |
| Tobacco smoking prevalence | 0.670** | 0.231 | 2.900 | 0.004 |
| Household air pollution | 0.393** | 0.105 | 3.743 | <0.001 |
| Ambient air pollution | -0.847** | 0.188 | -4.511 | <0.001 |
| Urban population | -0.162 | 0.13 | -1.249 | 0.213 |
| Human Development Index | 21.354 | 17.298 | 1.234 | 0.219 |

Note: Statistical significance level: * *p* < 0.05; ** *p* < 0.01.

**Supplementary Table 4.** Lagrange Multiplier (LM) tests, Spatial Effects and Fit Statistics for ordinary least squares (OLS), and Spatial Lag model.

| Risk factor | OLS | Spatial Lag Model |
| --- | --- | --- |
| ***Lagrange Multiplier (LM) tests*** | | |
| LM |  | 78.46** |
| Robust LM |  | 58.07** |
| ***Spatial Effects*** | | |
| ρ (Lag) |  | 0.441** |
| ***Fit Statistics*** | | |
| *R*^2^ | 0.34 | 0.39 |
| AIC | 1995.20 | 1993.24 |
| Moran's *I* of residual | 0.14** | -0.12 |

Note: Statistical significance level: * *p* < 0.05; ** *p* < 0.01. AIC, Akaike Information Criterion.
